# Is it all about Contact? Neurodegeneration as a “Protein Freeze Tag Game” Inside the Central Nervous System

**DOI:** 10.3389/fneur.2013.00075

**Published:** 2013-06-17

**Authors:** Tobias A. Mattei

**Affiliations:** ^1^Neurosurgery Department, University of Illinois at PeoriaPeoria, IL, USA

The so-called “prion hypothesis” for explaining spongiform encephalopathies is classically attributed to Prusiner, who in 1982 suggested that the scrapie agent was a proteinaceous infectious particle which would be resistant to known methods of nucleic acids inactivation (Prusiner, 1982).

Nevertheless such idea was not completely novel, once it has been already previously suggested (Gibbons and Hunter, 1967; Levine, 1972) that the scrapie agent might be devoid of disease-specific nucleic acid and, therefore, would have a different form of dissemination than known viral particles.

Earlier in 1968 the mathematician Griffith (1968) had proposed three distinct ways through which proteins might induce their own replication without the DNA/RNA machinery for nucleotide synthesis. Interestingly one of the explanations involved an analogy from the known necessity of the presence of initial atomic nuclei for gas condensation. Similarly, according to Griffith, in the protein level the “condensation nuclei” of a pre-existent polymer might (at least theoretically) be able to induce polymerization of other sub-units.

As protein polymerization with subsequent formation of deposit aggregates (such as beta-amyloid and neurofibrillary tangles) have been implied in the pathogenesis of several degenerative processes in the central nervous system (CNS), it was logical to suppose that the underlying pathogenesis of these diseases might have some similarity with the aforementioned “polymer hypothesis”, which has been postulated as the cause of propagation of misfolded proteins in spongiform encephalopathies.

In fact, several recent studies (Jucker and Walker, 2011; Hall and Patuto, 2012; Kanouchi et al., 2012) have suggested that the basic proteins implied in a variety of neurodegenerative diseases [like beta-amyloid and tau proteins in Alzheimer's disease (AD), α-synuclein in Parkinson Disease and dementia with Lewy bodies, polyglutamine proteins in Huntington's disease and spinocerebellar ataxia, and superoxide dismutase 1 in amyotrophic lateral sclerosis] may share important similarities with the mammalian prion protein (PrP^C^) involved in spongiform encephalopathies, such as the ability to translocate between neurons and further recruit normal proteins to aggregate.

The first suggestion of such possibility came from studies that demonstrated that a prion-like propagation mechanism of systemic amyloidoses occurred in animals through fecal transmission (Zhang et al., 2008). As several similarities exist between the pathophysiology of systemic and CNS amyloidoses, there has been a growing interest in the experimental evaluation of a possible protein-to-protein contact-induced transmission as the pathophysiological explanation for the progression of neurodegenerative diseases.

In a recent report Liu et al. (2012) described a new experimental protocol for the study of AD which involves a transgenic mouse that differentially expresses pathological human tau protein. In such animal model the authors demonstrated propagation of the pathological tau protein from the mesial portion of the entorhinal cortex into the CA1 region of the hippocampus and the dentate gyrus granule cells. Such findings strongly support a trans-synaptic mechanism of tau protein spreading between neurons along anatomically connected networks.

Actually, early experimental studies which investigated the mechanisms of propagation of AD had already shown that the injection of brain extracts from patients with AD into the brain of transgenic mice promoted the aggregation and deposition of β-amyloid in the injected brain (Kane et al., 2000).

Regarding the question about how would these initial abnormal proteins be able to spread the degenerative process to distant regions, it has been postulated that such cellular proteins could be released from neurons via vesicle mediated exocytosis or direct leakage through damaged cell membranes. The spatial propagation of these misfolded proteins would, therefore, explain the sequential symptomatic progression observed in the majority of the neurodegenerative diseases (Walker et al., 2002).

**Figure 1:**
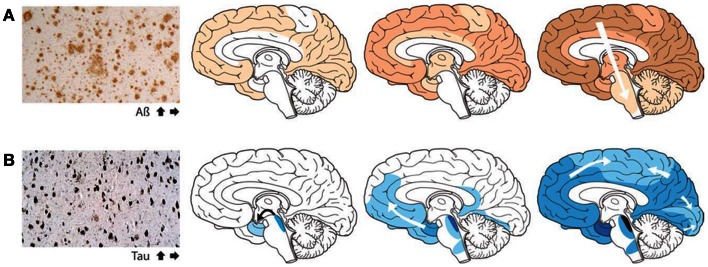
**The accumulation of misfolded proteins in Alzheimer disease follows a very characteristic and predictable pattern**. Cross-sectional autopsy studies indicate that β-amyloid plaques **(A)** first appear in the neocortex, followed by the allocortex and finally subcortical regions. In the brain, neurofibrillary tangles **(B)** occur first in the locus coeruleus and transentorhinal area, and then spread to the amygdala and interconnected neocortical brain regions. These relatively stereotyped patterns of expansion strongly suggest the involvement of neuronal transport mechanisms in the spread of such proteopathic seeds (Re-published with authorization from Jucker and Walker, 2011).

Although in some experiments involving artificial injection of brain extracts from patients with AD into the brains of mice, the induction of β-amyloid deposits was initially most evident within the injected area, recent cross-sectional autopsy studies have demonstrated that the accumulation of misfolded proteins follows a characteristic and predictable pattern of spatial progression in the brain of patients affected by AD (Jucker and Walker, 2011; Figure 1). These findings confirm the results of earlier studies which have shown sequential progression of neurofibrillary degeneration from the phylogenetically older mesial temporal regions to temporal cortical regions and finally to several other neocortical areas (Delacourte et al., 1999). Such dissemination was observed to occur first between non-contiguous (but axonally interconnected) regions, suggesting migration along already established neuronal pathways (Weller et al., 2008). Additionally a so-called “perivascular drainage pathway” has also been shown to possibly contribute to the observed dissemination (Klinge et al., 2006).

The experimental studies on such “prion-like” characteristics of the abnormal proteins involved in other neurodegenerative diseases is still their very initial phase, and the exact mechanism and routes through which such spreading might occur is still unknown. One important consequence which arises from the growing evidence for an infective role of the abnormal proteins related to neurodegeneration is an increasing attention to the possible role of the cerebrospinal fluid (CSF) circulation in the propagation (or alternatively in the clearance) of such abnormal proteins, rendering it as a possible valuable target for future therapies (Serot et al., 2011).

In summary, in the last decade several experimental and post-mortem autopsy studies have suggested that the abnormal proteins involved in several neurodegenerative diseases might present a “prion-like” behavior, in which the protein-to-protein contact would induce the further propagation of such abnormalities.

As already, mentioned such progression might also involve the active transport of such abnormal proteins to distant regions through axonal flow, perivascular spread, and, maybe, even through natural CSF circulation pathways. Despite the fact that, differently from the prionic proteins involved in spongiform encephalopathies, the inter-individual transmissibility of neurodegenerative diseases has never been reported, such new concept of disease progression by direct transmission through protein-to-protein contact present major implications for the current understanding of the pathophysiology of neurodegeneration. By emphasizing the likely relation between inter-cellular transmissibility and disease progression, such discoveries provide a new framework for experimental research in neurodegenerative diseases, as it promises to open further therapeutic avenues directed to inhibiting and eliminating such natural propagation processes.
